# Appendiceal Endosalpingiosis: A Rare Cause of Appendicitis-Like Presentation

**DOI:** 10.7759/cureus.117693

**Published:** 2026-10-09

**Authors:** Hamza Khan

**Affiliations:** 1 General and Colorectal Surgery, Royal Perth Bentley Group, Perth, AUS

**Keywords:** atypical appendicitis, diagnosis of acute appendicitis, emergency general surgery, endosalpingiosis, general surgery

## Abstract

We report a case of a 55-year-old female who presented to the emergency department with symptoms of appendicitis. Imaging demonstrated appendiceal mucocele and possible early appendicitis. She underwent a laparoscopic appendectomy and subsequent histopathology demonstrated irregular fibrosis of the appendiceal wall as a result of endosalpingiosis with secondary mucocele formation.

In this report, we present an exceedingly rare case of endosalpingiosis causing an appendicitis mimic. Although endosalpingiosis is typically considered a benign incidental finding, appendiceal involvement can mimic acute appendicitis, as in this case, and may be associated with an increased risk of gynecologic malignancy. The patient was followed up with the gynecological team due to this malignancy risk and is planned for laparoscopic oophorectomy, with the final histopathology diagnosis being appendiceal endosalpingiosis with secondary mucocele.

## Introduction

Appendicitis is inflammation of the vestigial appendix and is one of the most common causes of emergency abdominal surgery [1]. The appendix is located at the base of the cecum, near the ileocecal valve, where the taenia coli converge on the cecum [2]. The Global Burden of Disease reported 17 million incident cases of appendicitis in 2021, and it occurs most frequently in people in their 20s and 30s [3]. The classical pathogenesis of appendicitis is usually stated to be due to direct luminal obstruction. This can be caused by fecalith, lymphoid hyperplasia due to infection in young patients, fibrosis, or neoplasia. The classical history of appendicitis involves generalized abdominal pain that migrates to the right lower quadrant (RLQ), although this is only seen in 50-60% of patients with appendicitis. In summary, appendicitis is a very common surgical pathology with varying causes [4]. In this case, we describe fibrosis of the appendiceal wall as a result of endosalpingiosis in a 55-year-old female. This is a very rare cause of acute appendicitis.

## Case presentation

This case involves a 55-year-old female with a background history of endometriosis and hypertension (HTN). Her surgical history includes hiatal hernia and diaphragmatic hernia repair, C3-C4 and C4-C5 spinal fusion, and one C-section. The patient is postmenopausal but has a history of a single instance of postmenopausal bleeding. She is otherwise well and healthy, independent, lives at home with family, and does not smoke or drink alcohol.

She presented to our emergency department with a five-day history of right iliac fossa (RIF) pain that gradually increased in intensity. On the day of admission, the pain increased in intensity drastically, which prompted her to present to the emergency department. She described the pain as “grabbing” with no radiation. She reported that the pain was worse with movement and deep inspiration and did not have any urinary, bowel, or vaginal symptoms. She had no urinary, bowel, or vaginal symptoms and reported no nausea or vomiting. She was systemically well and afebrile on presentation.

On presentation, she was hemodynamically stable with no recorded fever; however, she was visibly uncomfortable with small movements. She was warm, well perfused, and euvolemic with normal heart and lung sounds. Her abdomen was soft, with tenderness in the RIF and right upper quadrant (RUQ), guarding, and a positive Rovsing’s sign. Given the findings on abdominal examination and clinical suspicion for acute appendicitis, she underwent a CT abdomen and pelvis.

Investigations

Her blood tests demonstrated a white cell count (WCC) of 7.06×10^9^/L (4.00-1.00×10^9^/L) and neutrophil count of 4.60×10^9^/L (2.00-7.50×10^9^/L). Her CRP was 8.4 mg/L (<5.0 mg/L) and lactate was 0.9 mmol/L (<2.0 mmol/L). The rest of her routine biomarkers were within normal ranges. She had surprisingly low inflammatory markers for a patient with possible appendicitis and five days of symptoms. Her CT abdomen and pelvis demonstrated a blind-ending tubular structure that communicates with the focal ovoid dilation at the tip, measuring up to 11 mm with internal low-attenuation (22 Hounsfield units {HU}) content, suggesting appendiceal mucocele without features of rupture (Figures 1, 2). There was no calcification or appendicolith, no significant peri-appendiceal fat stranding, free gas, or drainable collection.

**Figure 1:**
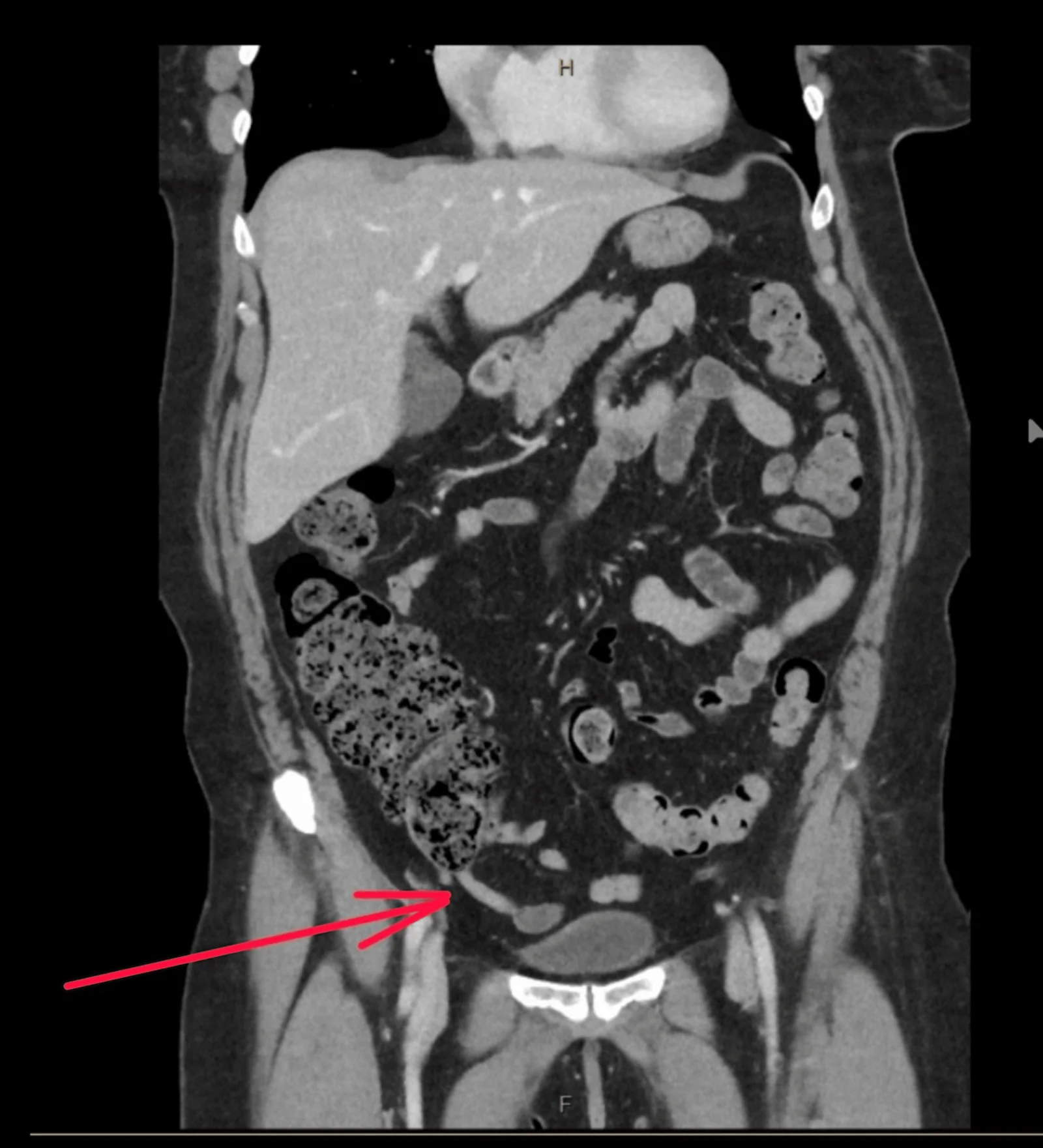
CT scan showing a blind-ending tubular structure communicating with focal ovoid dilation at tip (red arrow).

**Figure 2:**
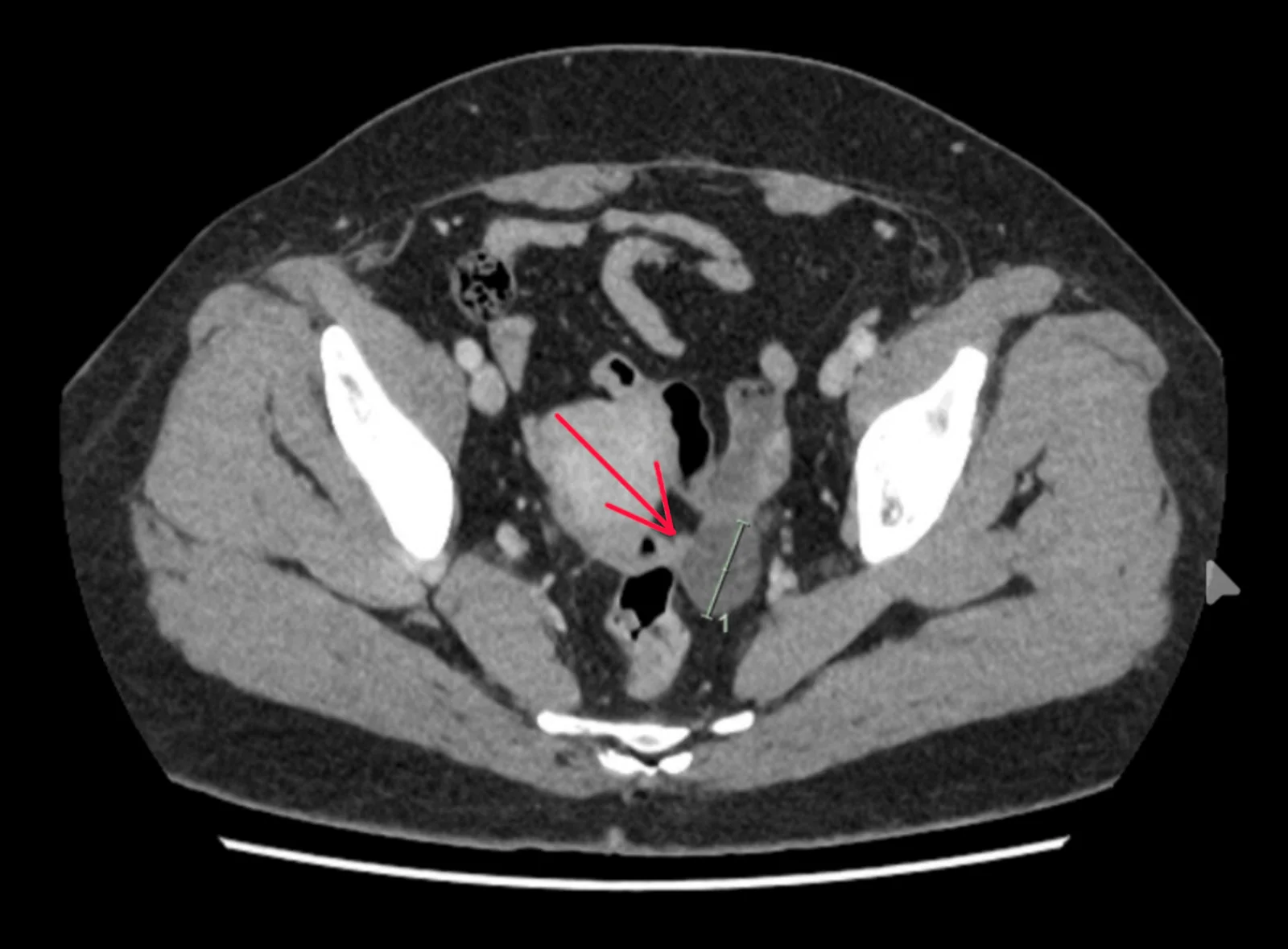
Focal ovoid dilation seen on CT at the time of presentation (red arrow).

Treatment

The patient was commenced on intravenous amoxicillin-clavulanate potassium, 2.2 g three times daily as per local hospital protocol, and underwent a laparoscopic appendectomy on the same day as her presentation. Intraoperative findings showed a mildly inflamed, non-perforated appendix with surrounding inflammatory tissue. There was no free fluid in the pelvis, and the base of the appendix was healthy with no spreading inflammation to the cecum. Care was taken not to spill any mucocele during the procedure. Extra precautions were taken, such as not letting the dissected appendix touch any body surface and applying diathermy to the appendiceal stump to ensure an adequate seal was formed.

Follow-up

The patient received her histopathology results during a telephone consultation three weeks after surgery. Following her operation, the patient was well and returned to daily life with the restrictions of not lifting more than 5 kg for six weeks. She returned to the ED with abdominal pain but was discharged with safety netting after a normal repeat CT scan and blood tests. She had no postoperative complications. Her histopathology results are detailed below.

Macroscopic

The appendix measured 38 mm in length and 15 mm in diameter. The attached mesoappendix measured 14 mm. The serosal surface was gray-tan in color, with a glossy appearance. The lumen contained a transparent, gelatinous-like material. The wall thickness measured up to 4 mm.

Microscopic

There was irregular fibrosis of the appendiceal wall as a result of endosalpingiosis. There was secondary mucocele formation. There were no features of malignancy. No active inflammation was noted. The mucosa at the resection margin was unremarkable and consistent with normal large bowel mucosa.

Although the histopathology did not show active inflammation, the case was described as acute appendicitis given the clinical presentation, which was consistent with the classical presentation of appendicitis. She was followed up in the gynecological clinic given the histopathology findings and was booked for a laparoscopic oophorectomy.

## Discussion

Appendiceal mucinous lesions are a relatively rare finding characterized by a distended, mucus-filled, or ruptured appendix. There are an estimated 3500 cases of appendiceal mucinous neoplasms diagnosed annually in the United States [5]. Appendiceal mucinous lesions have a slight predominance in females and are usually diagnosed in patients in their 50s and 60s [6].

The development of appendiceal mucocele is usually due to obstruction and increased accumulation of mucus within the appendix lumen. The etiology can range from a simple retention cyst to a malignant neoplasm. Diagnosis of a mucocele should be made promptly, as a ruptured neoplastic mucocele can lead to development of pseudomyxoma peritonei, colloquially known as “jelly belly.” This can result in disseminated mucin-producing cells throughout the peritoneal cavity, which can drastically worsen patient prognosis and outcomes [7].

Appendiceal mucinous lesions can be classified broadly into two categories. Non-neoplastic lesions involve simple mucoceles or retention cysts. These are characterized by degenerative epithelial changes due to obstruction and distension, such as in the case of a fecalith, without evidence of mucosal hyperplasia or neoplasia. Neoplastic appendiceal mucinous lesions can be subdivided into serrated polyps, mucinous appendiceal neoplasms, and mucinous adenocarcinoma of the appendix [8].

Endosalpingiosis is the presence of epithelium that resembles the fallopian tube endothelium in a non-native area, including pelvic organs, ovaries, pelvic peritoneum, bladder, or fallopian tube serosa [9,10]. It is not well studied, and definitive diagnosis is only made after surgical biopsy [11]. It is often an incidental finding during pelvic surgery.

There have been studies that describe the association between endosalpingiosis and ovarian cancer. A study of 110 cases of endosalpingiosis found endometrial adenocarcinoma in 16% of cases and borderline ovarian tumors in 7% of cases. In the patients with borderline ovarian tumors, endosalpingiosis was found on the ovary (37%), fallopian tube (21%), omentum (9%), and uterine serosa (7%) [12]. Another study of 838 cases reported that patients with endosalpingiosis were more likely to have serous borderline, invasive mucinous, and clear cell ovarian tumors when compared to a population control group [13]. A large retrospective study of 2490 patients with a histological diagnosis of endosalpingiosis had a higher incidence of ovarian cancer than the control group [14]. While endosalpingiosis is not well studied, these few studies establish a potential link between endosalpingiosis and increased malignancy risk, indicating that it is a condition of consequence and not merely an incidental finding. Although a definite causal relationship has not been established, an association between endosalpingiosis and malignancy has been reported.

Endosalpingiosis of the appendix is significantly rarer. Esselen et al. described only 20 cases of appendiceal endosalpingiosis from 1998 to 2013 [13]. Of these cases, Tudor et al. described two cases of women in their 40s-50s presenting with classical appendicitis symptoms [15]. Both of these cases had normal inflammatory markers, with histopathology confirming the diagnosis of endosalpingiosis. Cajigas and Axiotis described a similar case to ours but with mild leukocytosis and a normal-appearing appendix intraoperatively [16]. Other reports describe similar presentations, indicating that these patients often present with presumed mild appendicitis and the diagnosis is only confirmed after histopathology.

There is very limited research regarding appendicitis secondary to endosalpingiosis. However, there is an association with increased malignancy risk, especially ovarian cancer. Esselen et al. also described an increased risk of ovarian invasive mucinous cancer in patients with endosalpingiosis [13]. Given the current literature, cases such as these should be closely followed up by the gynecological team for further malignancy workup. Surgeons should take care to chase the histopathology in patients who have presumed mucinous appendicitis and refer to the appropriate team to avoid missing a potential gynecologic malignancy.

Limitations

The main limitation of this case is the lack of literature on endosalpingiosis causing an appendicitis mimic. As mentioned, only 20 cases have been described in the literature. Furthermore, an association between endosalpingiosis and gynecological malignancy has been demonstrated, but a true causation cannot be proven. Lastly, this patient is undergoing evaluation by the gynecological team; therefore, long-term follow-up was not available at the time of publication.

## Conclusions

Endosalpingiosis can be a rare cause of appendicitis mimic and can often present with classical appendicitis symptoms but with minimal inflammatory response on blood tests, imaging, or histopathology. Endosalpingiosis was previously thought to be a benign incidental finding, but recent studies have demonstrated an association with increased malignancy risk. If found intraoperatively or on histopathology postoperatively, the surgical team should refer to the gynecological team for further workup and management of potential malignancy. The main finding of this case is that endosalpingiosis has been associated with increased gynecological malignancy risk and can mimic appendicitis and cause mucocele formation.

## Appendices

Patient experience

I have a history of endometriosis and have always had very painful menstrual cycles and right-sided pelvic pain. I had postmenopausal bleeding, and thickened endometrium of 9 mm and a hypoechoic lesion within the endometrium measuring 9x7 mm with an echogenic rim. I was referred to a gynecologist, who did a hysteroscopy and endometrial biopsy. The report came back unremarkable; the endometrium was atrophic, no curettings were obtained, and there was no evidence of fibroids or polyps.

I am always bloated and have a dull, aching pain most of the time in the right pelvic area. Leading up to the appendix removal, I was doing housework and experienced right-sided pain so bad I couldn't walk, and my son had to help me to lie down. I thought I must have twisted the wrong way or had pulled something. So I put a wheat bag on it. Pain was at least a 10, a grabbing-like pain, and intense, and if I lay down and did not move, it was okay. I tried to put a pillow under my legs; again, pain was at a 10. I rested that afternoon; the next morning, pain was at a 10 when I would sit down, get up, or walk. Driving was ok but again, when I tried to get out and stand, the pain was back. Over the next few days, the pain was intermittent but immediately 10 when I went from sitting to standing. One night, the pain woke me up; I couldn't get up or walk, and then I went to the hospital.

I am still experiencing bloating, bleeding, and right-sided pelvic pain, so I made an appointment with my GP, who advised me to go back to the ED for follow-up, which I did. I was then referred to the gynecological team. I had an appointment with a gynecologist who suggested I have a laparoscopic oophorectomy. However, I went back to my specialist, who thinks that, given my history, it would be better to have a full hysterectomy including fallopian tubes and ovaries. I am still deciding the best option, but after reading your research, I will probably get the full hysterectomy, including the fallopian tubes and ovaries, due to the risk of malignancy from the appendix findings.
